# Is the p-value properly interpreted by critical care professionals? Online survey

**DOI:** 10.5935/0103-507X.20210009

**Published:** 2021

**Authors:** Mauro Federico Andreu, Ladislao Pablo Diaz Ballve, Daniel Héctor Verdecchia, Agustina Maria Monzón, Tatiana Dias de Carvalho

**Affiliations:** 1 Licenciatura en Kinesiología y Fisiatría, Departamento de Ciencias de la Salud, Universidad Nacional de La Matanza - San Justo, Buenos Aires, Argentina.; 2 Intensive Care Unit, Hospital General de Agudos Donacion Santojanni - Buenos Aires, Argentina.; 3 Hospital Nacional Profesor Alejandro Posadas - Buenos Aires, Argentina.

**Keywords:** Biostatistics, Biomedical research/statistics & numerical data, Data interpretation, statistical, Hypothesis testing, Evidence-based medicine, Prevalence, Bioestatística, Pesquisa biomédica/estatística & dados numéricos, Interpretação estatística de dados, Testes de hipóteses, Medicina baseada em evidências, Prevalência

## Abstract

**Objective:**

To determine the prevalence of and risk factors for insufficient knowledge related to p-values among critical care physicians and respiratory therapists in Argentina.

**Methods:**

This cross-sectional online survey contained 25 questions about respondents’ characteristics, self-perception and p-value knowledge (theory and practice). Descriptive and multivariable logistic regression analyses were conducted.

**Results:**

Three hundred seventy-six respondents were analyzed. Two hundred thirty-seven respondents (63.1%) did not know about p-values. According to the multivariable logistic regression analysis, a lack of training on scientific research methodology (adjusted OR 2.50; 95%CI 1.37 - 4.53; p = 0.003) and the amount of reading (< 6 scientific articles per year; adjusted OR 3.27; 95%CI 1.67 - 6.40; p = 0.001) were found to be independently associated with the respondents’ lack of p-value knowledge.

**Conclusion:**

The prevalence of insufficient knowledge regarding p-values among critical care physicians and respiratory therapists in Argentina was 63%. A lack of training on scientific research methodology and the amount of reading (< 6 scientific articles per year) were found to be independently associated with the respondents’ lack of p-value knowledge.

## Abstract (pt)

**Objetivo:**

Determinar a prevalência e os fatores de risco para conhecimento insuficiente sobre valores de p entre médicos e terapeutas respiratórios atuantes em terapia intensiva na Argentina.

**Métodos:**

Levantamento transversal on-line com 25 questões relativas às características dos participantes, autopercepção e conhecimento sobre valores de p (teoria e prática). Realizaram-se análises de estatística descritiva e regressão logística multivariada.

**Resultados:**

Analisaram-se 376 participantes. Não tinham conhecimento a respeito dos valores de p 237 participantes (63,1%). Segundo análise de regressão logística multivariada, falta de treinamento em metodologia científica (RC ajustadas 2,50; IC95% 1,37 - 4,53; p = 0,003) e a quantidade de leitura (< 6 artigos científicos por ano; RC ajustadas 3,27; IC95% 1,67 - 6,40; p = 0,001) foram identificados como independentemente associados com a falta de conhecimento sobre valores de p por parte dos participantes.

**Conclusão:**

A prevalência de conhecimento insuficiente com relação a valores de p entre médicos e terapeutas respiratórios na Argentina foi de 63%. Falta de treinamento em metodologia científica e quantidade de leitura (< 6 artigos científicos por ano) foram identificados como independentemente associados com a falta de conhecimento sobre valores de p por parte dos participantes.

## INTRODUCTION

Healthcare professionals must rely on updated clinical information to practice evidence-based medicine (EBM).^(1)^ To address their clinical questions, healthcare professionals need to critically appraise the design and procedure of the studies and interpret the results.^(2)^ Null hypothesis (H0) significance testing based on p-values-indicators used to reject or not reject null hypotheses-is the primary technique for drawing conclusions from data in many health disciplines.^(3)^

Several survey studies have demonstrated that a large number of healthcare professionals are unable to understand and interpret statistical results appropriately.^(4-7)^ Horton et al. reported that many health professionals have increased difficulty because increasingly complicated statistical methods are being reported in the medical literature, and thus, these professionals may be able to understand the analysis and interpretation of results in only 21% of research articles.^(8)^

Informally, a p-value is the probability under a specified statistical model that a statistical summary of the data (e.g., the sample mean difference between two groups being compared) would be equal to or more extreme than its observed value.^(9)^ The most common misconceptions about the p-value are the inverse probability fallacy, replication fallacy, clinical or practical significance fallacy, and effect size fallacy.^(10-13)^

The “inverse probability fallacy” is the false belief that the p-value indicates the probability that H0 is true, given certain results [P (H0/results)]. Essentially, it means confusing the probability of the result, assuming that the null hypothesis is true [P (results/H0)], with the probability of the null hypothesis, given certain data [P (H0/results)].^(14)^

The second misconception is called the “replication fallacy”, which is the belief that the p-value is the degree of replicability of the result, and its complement, 1-p, is frequently misinterpreted as the probability a result will be replicated.^(10,13)^ That is, the belief that result with a p-value of 0.05 means that 95 times out of 100, the statistically significant results obtained in a study will be the same in future research.^(15)^ However, p-values provide only very little information about what is likely to happen upon replication, and they may differ upon replication simply because of sampling variability.^(3)^

The false belief that the p-value provides direct information about the effect size is called the “effect size” fallacy.^(16)^ Researchers believe that the smaller the p-value is, the larger the effect size is.^(12,17)^

The last misconception is called the “clinical or practical significance” fallacy, which relates statistical significance to the importance of the effect size.^(10)^ A statistically significant result, however, may lack clinical significance, and vice versa; therefore, the clinical or practical significance of the findings should be described by an expert in the field and not presented by statistics alone.^(11)^

Despite the important role played by statistical interpretation and critical appraisal of published studies in the practice of EBM, there is not enough evidence regarding critical care professionals’ knowledge of the topic.

The objective of this study was to determine the prevalence of and risk factors for insufficient p-value knowledge among critical care physicians and respiratory therapists in Argentina.

## METHODS

This is an observational cross-sectional survey study conducted between August 30 and November 30, 2018. Informed Consent was not required since participation was voluntary and anonymous. The protocol study was approved by the *Hospital Nacional Profesor Alejandro Posadas* Ethics Committee (312 EmnPeS0/19).

We included healthcare professionals in the field of cardiorespiratory care in our analysis. Professionals not working in Argentina and those who quit the survey before section B (filter question) were excluded from our analyses.

### Pilot testing

Before the study, a pilot test was conducted to assess the viability and feasibility of the survey. The survey was administered to 42 healthcare professionals, and the time required to answer the questions was recorded. We also asked each professional to report whether the survey, or a specific question, presented any difficulties. Forty (95.2%) respondents stated that the survey was clear and that they understood its objective. Thirty-seven (88.1%) understood all the questions. Three participants had difficulties with question 19, and two participants had difficulties with question 5. With respect to the degree of difficulty, seven respondents (16.7%) considered that the survey was very easy; five (11.9%) considered the survey easy; 13 (31%) considered the survey moderate; 15 (35.7%) considered the survey difficult; and two (2.4%) considered the survey very difficult. The median time to respond to the survey was 6.5 (5 - 8) minutes.

### Data collection

Through convenience and no probabilistic sampling, professionals were invited to participate via email and social networks. The invitation included the objective of the study and a link to access the survey online through the SurveyMonkey® tool (https://es.surveymonkey.com/r/valorp).

### Instrument

The survey contained 25 questions divided into three sections (Appendix 1).

The first section (A) consisted of 13 nominal and ordinal questions about the respondents’ professional characteristics, such as background, academic education, and experience in scientific reading.

The second section (B) consisted of one nominal dichotomous (Yes/No) question pertaining to the respondents’ self-perception about their p-value knowledge. If the answer was negative, the survey ended.

Finally, the third section (C) consisted of 11 nominal (T/F) questions (True/False/Do not know) about p-values: 6 theory questions, 4 practice interpretation questions, and 1 definition question (Appendix 1).

Questions in section C were administered in a random order.

### Primary outcome measure

The lack of p-value knowledge was the main outcome of the study. Respondents who stated they did not know about p-values (a “no” answer to the section B question) or those who did not reach the required score in any of the two categories (theory or practice) were considered “unknowledgeable about p-values”. Those who quit the survey in section C without reaching the required threshold in at least one of the two categories (theory or practice) were also considered “unknowledgeable about p-values”.

### Statistical analysis

Categorical variables are presented as numbers and percentages. Continuous variables with a normal distribution are presented as the mean and standard deviation. Nonnormally distributed variables are presented as medians and interquartile ranges. The distribution of continuous variables was assessed using the Kolmogorov-Smirnov test.

The test for a difference in proportions was performed to compare nominal variables between categories.

The main outcome was lack of p-value knowledge (theory and/or practice). P-value knowledge questions were grouped into theory questions (15, 16, 19, 20, 21, and 22) and practice questions (17, 18, 23, and 24). The respondent was considered to have sufficient theoretical knowledge if at least 4 out of 6 theory questions (67%) were correctly answered. The respondent was considered to have sufficient practical knowledge if at least 3 out of 4 practice questions (75%) were correctly answered. The respondent was considered to know about p-values if the required score was reached in either of the two categories.

The associations between p-value knowledge and other variables were determined via univariate analysis. The odds ratios (OR) and their corresponding 95% confidence intervals (95%CI) were reported. Variables with a p-value < 0.15 were included in the multivariable logistic regression model to identify those that were independently associated with p-value knowledge. A backward conditional stepwise (Wald) method was used. A p-value < 0.05 was considered significant. Statistical analysis was performed using IBM Statistical Package for Social Sciences (SPSS), v. 22.0, software for Macintosh (IBM Corp., Armonk, NY, United States).

## RESULTS

A total of 896 surveys were collected; 520 were excluded because the eligibility criteria were not met.

A total of 376 surveys were analyzed: 210 (55.9%) participants were physicians, and 166 (44.1%) were respiratory therapists. The characteristics of the sample are detailed in table 1. Only 139 (37.0%) respondents answered the p-value questions satisfactorily (at least 4 correct theory responses and/or 3 correct practice responses).

**Table 1:** Sample characteristics

Variables	
Age (years)	36 (29 - 48)
Male sex	195 (51.9)
Years since graduation from degree program	12 (7 - 23)
Degree/specialization	
Physician/cardiologist	20 (5.3)
Physician/intensive therapist	100 (26.6)
Physician/pulmonologist	90 (23.9)
Respiratory therapist	166 (44.1)
Education in a private university	45 (12)
Complete level of training	
Courses	166 (44.1)
Residency, scholarship	246 (65.4)
Specialization or advanced course	246 (65.4)
Master´s Degree program	24 (6.4)
Doctorate Degree program	14 (3.7)
Training on scientific research methodology	83 (22.1)
Read 6 or more scientific articles per year	73 (19.4)
Consider the language of publication a barrier to reading scientific articles	226 (60.1)
Have authored a scientific article	202 (53.7)

Results expressed as median (interquartile range) or n (%).

Two hundred thirty-seven respondents did not understand p-values [63.1% (95%CI 58.0% - 67.7%)]. Of these respondents, 47 (12.5%) reported that they did not understand p-values, and 190 (50.5%) reported that they did understand p-values even though they did not reach the cutoff scores for either of the knowledge categories (theory and practice). The results of sections B and C (questions 14 through 24) are summarized in table 2.

**Table 2:** Survey results

**Do you know what the p-value is?**	n = 47/376 (12.5%) respondents answered "No" and were considered unknowledgeable about the p-value (end of survey)									
	n = 329/376 (87.5%) respondents answered "Yes" and continued with p-value questions									
**p-value questions**	**Theory questions**					**Practice questions**				
	The p-value is a probability	A nonsignificant p-value (p > 0.05) indicates that the null hypothesis is true	The p-value indicates the probability that the null hypothesis is true given the results of our study	A nonsignificant p-value (p > 0.05) indicates we should accept the null hypothesis	If we obtain a significant p-value (p < 0.05), we should reject the null hypothesis	The p-value obtained (p = 0.02) indicates the probability of obtaining similar results if the same study is repeated with a similar sample	Um valor não significante de p (p > 0,05) indica que o efeito do tratamento em análise não é clinicamente importante	A nonsignificant p-value (p > 0.05) indicates that both treatments are similar	A statistically significant result (p < 0.05) indicates that the effect of the treatment under analysis is clinically important	The p-value observed in our study was significant (p = 0.02). This confirms that the effect of the treatment was higher than that observed in a similar study with a p-value = 0.04
**Correct, n (%)**	251 (66.8)	132 (35.1)	68 (18.1)	115 (30.6)	169 (44.9)	80 (21.3)	127 (33.8)	132 (35.1)	102 (27.1)	147 (39.1)
**Incorrect, n (%)**	59 (15.7)	145(38.6)	171 (45.5)	126 (33.5)	72 (19.1)	154 (41.0)	162 (43.1)	143 (38.0)	154 (41.0)	89 (23.7)
**I do not know, n (%)**	10 (2.7)	27(7.2)	46 (12.2)	39 (10.4)	32 (8.5)	36 (9.6)	9 (2.4)	17 (4.5)	12 (3.2)	30 (8.0)
**Overall result**			n = 237/376; 63,0% (95%CI 58,0% - 67,7%) know neither theory nor practice							
			n = 139/376; 37,0% (95%CI 32,2% - 41,9) know theory and/or practice							
			n = 28/376; 7.4% (95%CI 5,2% - 10,5%) know theory and practice							
			n = 69/376; 18,3% (95%CI 14,7% - 22,5%) know theory							
			n = 84/376; 22,3% (95%CI 18,4% - 26,8%) know practice							

95%CI - 95% of confidence interval.

**Figure 1:**
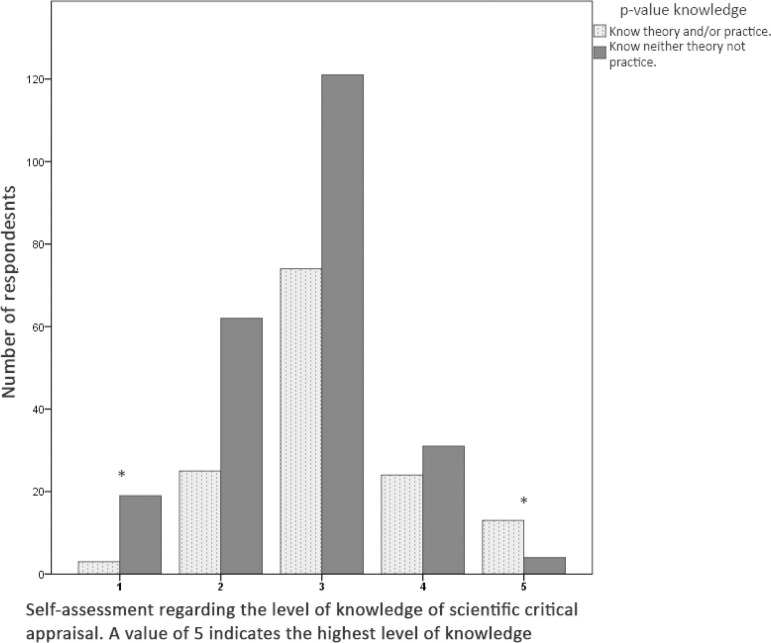
Overall p-value knowledge scores according to respondents’ self assessment regarding scientific critical appraisal.

Respondents’ self-assessment regarding “critical appraisal of a scientific article” and its association with the overall survey result (understanding or not understanding p-values) are detailed in figure 1 (p < 0.001). Differences were only observed between the respondents who understood p-values (the highest scores) and all other participants as well as between those who did not understand p-values (the lowest scores of the scale) and all other participants (p = 0.019 and p = 0.005, respectively).

* Statistical significance for the comparison between “understanding” and “not understanding” p-values.

In question 25, respondents had to choose the correct p-value definition (item c, “both options are correct”). Only 104 of 376 respondents (27.6%) answered this item correctly.

The univariate and multivariable binary logistic regression models are detailed in table 3. According to the multivariable logistic regression analysis, a lack of training on scientific research methodology (adjusted OR 2.50 [95%CI 1.37 - 4.53], p = 0.003) and the amount of reading (< 6 scientific articles per year) (adjusted OR 3.27 [95%CI 1.67 - 6.40], p = 0.001) were found to be independently associated with the respondents’ lack of p-value knowledge.

**Table 3:** Univariate and multivariable binary logistic regression analysis

Variables	OR (95% CI)	p value	Adjusted OR (95%CI)	p value
Age	1.01 (0.99 - 1.02)	0.26		
Male sex	0.76 (0.50 - 1.16)	0.21		
Years since graduation	1.00 (0.98 - 1.01)	0.99		
Private university	0.66 (0.33 - 1.31)	0.23		
Highest level completed in postgraduate education				
Training course	0.88 (0.58 - 1.35)	0.57		
Residency, scholarship	0.73 (0.47 - 1.15)	0.17		
Specialization or advanced course	1.22 (0.79 - 1.89)	0.38		
Master´s Degree program	1.22 (0.79 - 1.89)	0.18		
Doctorate Degree program	0.42 (0.14 - 1.25)	0.12	0.64 (0.20 - 2.00)	0.44
Lack of training on scientific research methodology	2.77 (1.55 - 4.95)	0.001	2.50 (1.37 - 4.53)	0.003
Read < 6 articles per year	3.67 (1.896 - 7.09)	< 0.001	3.27 (1.67 - 6.40)	0.001
Consider the language of publication a barrier to reading scientific articles	1.43 (0.93 - 2.19)	0.1	1.13 (0.72 - 1.79)	0.58
Low self-assessment regarding scientific reading	2.09 (1.24 - 3.5)	0.005	1.63 (0.95 - 2.80)	0.073
Have authored a scientific article	0.51 (0.33 - 0.78)	0.002	0.72 (0.45 - 1.15)	0.17

OR - odds ratio; 95%CI - 95% of confidence interval.

## DISCUSSION

Our main finding was a high prevalence of insufficient p-value knowledge among critical care physicians and respiratory therapists. These findings are in line with the results of prior studies. Such results revealed that a high percentage of healthcare professionals experienced difficulties in understanding and interpreting p-values.^(18-21)^

According to a survey conducted by Badenes-Ribera et al. among Spanish psychology professors, many university professors did not know how to correctly interpret p-values.^(22)^ The authors conducted a similar survey among Italian and Chilean psychology university students and observed that a percentage of the respondents were not able to interpret p-values.^(14)^

Msaouel et al. performed a multi-institutional survey of Greek medical residents about basic statistical concepts.^(20)^ The results showed that a large number of medical residents were unable to correctly interpret the concepts that are commonly found in the medical literature. Susarla and Redett also assessed the knowledge, attitudes and confidence with biostatistics in a similar population.^(23)^ The authors concluded that residents place a high degree of importance on biostatistics knowledge, but they have only a fair understanding of core statistical concepts.

In accordance with our study, two factors were found to be independently associated with the respondents’ lack of p-value knowledge: a lack of training on scientific research methodology and the amount of reading (< 6 scientific articles per year). These results are consistent with the literature.^(24)^

In our study, we also noticed that being trained in research methodology does not prevent professionals from misinterpreting p-values. The assumption that training prevents incorrect interpretations is a false belief that could be spread among less experienced or trainee colleagues.^(25)^

A study that assessed medical residents’ attitudes and confidence with epidemiology and biostatistics concluded that being trained in biostatistics and reading a higher number of journals in statistics and epidemiology on a monthly basis were associated with a positive attitude towards biostatistics and increased confidence with statistical concepts.^(23)^ Similarly, our results indicates that professionals who read more than 6 scientific articles per year had higher levels of p-value knowledge.

The lack of p-value knowledge was more prevalent with respect to theoretical knowledge than practical knowledge. This may be because when healthcare professionals read scientific articles, they do not usually apply a *sine qua non* probabilistic interpretation of p-values. Such results only require the reader to routinely apply the p < alpha rule. Therefore, statistical interpretation is only based on the valuation of the p-value compared to the alpha value.^(26)^ This presumption seems to be based on the results obtained for the question about p-values. Although there was no statistically significant difference between the professionals who understood p-values and those who did not, a high number of respondents could not provide a correct definition.^(9,27)^

Respondents’ self-assessment regarding critical appraisal should be highlighted. Respondents who reported having remarkable critical appraisal skills (five points) responded to the survey correctly. Likewise, respondents who reported having poor critical appraisal skills (one point) also showed low levels of p-value knowledge. However, it is noteworthy that a large percentage of respondents who reported high critical appraisal skills (three or four points) failed to reach the cutoff scores for p-value knowledgeable. This finding could be due to the existing contradiction between poor training in statistics and the oversized importance placed on the p-value in medical publications.

The most common misconceptions of the p-value are the “fallacies” that may seriously jeopardize the correct interpretation of results.^(10-13)^ In agreement with our results, Msaouel et al. also observed that medical residents are especially prone to the *gambler fallacy* bias. This is caused by the erroneous belief according to which an event is more likely to occur if it has not previously occurred and vice versa. This bias may undermine clinical judgment and medical decision making.^(20)^

P-values may be misinterpreted due to multiple factors, such as the results and publication biases observed in the literature. Results bias is the phenomenon of authors reporting only satisfactory results. On the other hand, publication bias is the phenomenon of scientific journals accepting only articles with statistically significant results and rejecting articles with nonsignificant results.^(28-31)^

More than 12% of the respondents reported that they did not understand p-values. This probably indicates that some professionals do not read scientific articles. It is therefore necessary to improve training in this field to ensure highquality knowledge.^(25)^ Proper systematic training in biostatistics is required to debias professionals and ensure that they are proficient in understanding and communicating statistical information.^(20)^

This study has some limitations. First, those respondents who quit in section C were considered to “lack p-value knowledge”. Therefore, we might have overestimated the prevalence of insufficient knowledge, since these respondents may have finished the survey and reached the cutoff scores for p-value knowledge. Similarly, we have considered participants who answered negatively to question 14 to “lack p-value knowledge” without allowing them to continue with the questions in section C. The reason for excluding these participants was justified because it could have resulted in a greater number of dropouts and incomplete answers due to the survey length and the possibility of participants providing random answers just to complete the survey. This could be another factor that jeopardizes the validity of the “lack of p-value knowledge” estimate.

Second, we arbitrarily grouped questions into theory and practice knowledge and arbitrarily determined the cutoff scores to define a lack of knowledge. However, even if the questions were posed by the authors, the content assessed in each of them was based on prior studies.^(14,22)^ Moreover, to avoid random responses, we added the option “I do not know”. Another limitation of this study is the fact that we did not use a validated instrument, but to minimize this limitation, we conducted a pilot test in which virtually 90% of the respondents answered that they understood all the questions.

This is the first study to report the level of p-value knowledge among critical care physicians and respiratory therapists in Argentina. According to the results, we consider that training in critical appraisal should be included in the curricula of first-degree programs, with specialization in scientific literature reading and interpretation. Furthermore, healthcare professors should encourage their students to attend and participate in scientific activities.

## CONCLUSION

The overall prevalence of insufficient p-value knowledge among critical care physicians and respiratory therapists in Argentina was 63%. Two factors were found to be independently associated with the respondents’ lack of p-value knowledge: a lack of training on scientific research methodology and the amount of reading (< 6 scientific articles per year).

## Appendix 1- Survey.

**Appendix 1:** Survey.

A. Respondent characteristics	**B. Self-perception about your p-value knowledge**
	
1. Date of birth:	14. Do you know what the p-value is?
	a) Yes b) No (end of survey).
2. Sex:	
a) Male. b) Female.	**C. P-value questions**
	
3. Country of residence:	15. The p-value is a probability
a) Argentina. b) Other (specify).	a) True. b) False. c) I do not know.
	
4. Year of graduation	Imagine we are conducting a study in which two treatment groups are being compared, and we define a p-value < 0.05 (type I error or α < 5%) as "statistical significance". Based on this premise, mark whether the following statements about p-value interpretation are true or false.
	
5. Discipline:	16. A nonsignificant p-value (p > 0.05) indicates that the null hypothesis is true.
a) Medicine b) Respiratory therapy. c) Nursing. d) Other (specify).	a) True. b) False. c) I do not know.
	
6. Main area of professional practice:	17. A nonsignificant p-value (p > 0.05) indicates that the effect of the treatment under analysis is not clinically important.
	a) True. b) False. c) I do not know.
7. You completed your degree program in a:	
a) Public university. b) Private university.	18. A nonsignificant p-value (p > 0.05) indicates that both treatments are similar.
	a) True. b) False. c) I do not know.
8. Indicate the level of education you have completed:	
a) Training courses. b) Residency/scholarship. c) Fellowship. d) Specialization/advanced course. e) Master´s Degree - Program. f) Doctorate Degree - Program. g) Post-Doctorate.	19. The p-value indicates the probability that the null hypothesis is true given the results of our study.
	a) True. b) False. c) I do not know.

	20. A nonsignificant p-value (p > 0.05) indicates we should accept the null hypothesis.
	a) True. b) False. c) I do not know.
	
9. Have you received training in research methodology or scientific critical appraisal, either as a course or complement to the curriculum?	21. If we obtain a significant p-value (p < 0.05), we should reject the null hypothesis.
	a) True. b) False. c) I do not know.

a) Yes. b) No.	22. The p-value obtained (p = 0.02) indicates the probability of obtaining similar results if the same study is repeated with a similar sample.
	a) True. b) False. c) I do not know.
	
10. How many scientific articles have you aproximately read in the last year?	23. A statistically significant result (p<0.05) indicates that the effect of the treatment under analysis is clinically important.
a) I have not read any scientific articles in the last year. b) 1 to 5. c) 6 to 12. d) More than 12.	a) True. b) False. c) I do not know.
	
11. In your opinion, do you think that language is a barrier to reading scientific articles?	24. The p-value observed in our study was significant (p = 0.02). This confirms that the effect of the treatment was higher than that observed in a similar study with a p-value = 0.04.
a) Yes. b) No.	a) True. b) False. c) I do not know.
	
12. Have you authored an article published in a scientific	25. Which of the following statements is the definition of the p-value?
a) Yes. b) No.	a) The p-value is the probability of obtaining a result that is equal to, or more extreme than, the result observed.
	b) The p-value indicates to what degree data are not consistent with the null hypothesis.
13. On a 5-point scale, what level of knowledge do you think you have about scientific critical appraisal (5 represents the highest level of knowledge)?	c) Both options are correct.
	d) I do not know.
